# Improving Eye–Computer Interaction Interface Design: Ergonomic Investigations of the Optimum Target Size and Gaze-triggering Dwell Time

**DOI:** 10.16910/jemr.12.3.8

**Published:** 2020-09-25

**Authors:** Ya-feng Niu, Yue Gao, Ya-ting Zhang, Cheng-qi Xue, Li-xin Yang

**Affiliations:** School of Mechanical Engineering, Southeast University, China

**Keywords:** ECI, dwell time, target size, usability, ergonomics

## Abstract

Interactive feedback of interface elements and low level of spatial accuracy are two main key points for the interaction research in the Eye-computer interaction system. This study tried to solve these two problems from the perspective of human–computer interactions and ergonomics. Two experiments were conducted to explore the optimum target size and gaze-triggering dwell time of the eye–computer interaction (ECI) system. Experimental Series 1 was used as the pre-experiment to identify the size that has a greater task completion rate. Experimental Series 2 was used as the main experiment to investigate the optimum gaze-triggering dwell time by using a comprehensive evaluation of the task completion rate, reaction time, and NASA-TLX (Task Load Index). In Experimental Series 1, the optimal element size was determined to be 256 × 256p x 2. The conclusion of Experimental Series 2 was that when the dwell time is set to 600 ms, the efficiency of the interface is the highest, and the task load of subjects is minimal as well. Finally, the results of Experiment Series 1 and 2 have positive effects on improving the usability of the interface. The optimal control size and the optimal dwell time obtained from the experiments have certain reference and application value for interface design and software development of the ECI system.

## Introduction

**Figure 1:**
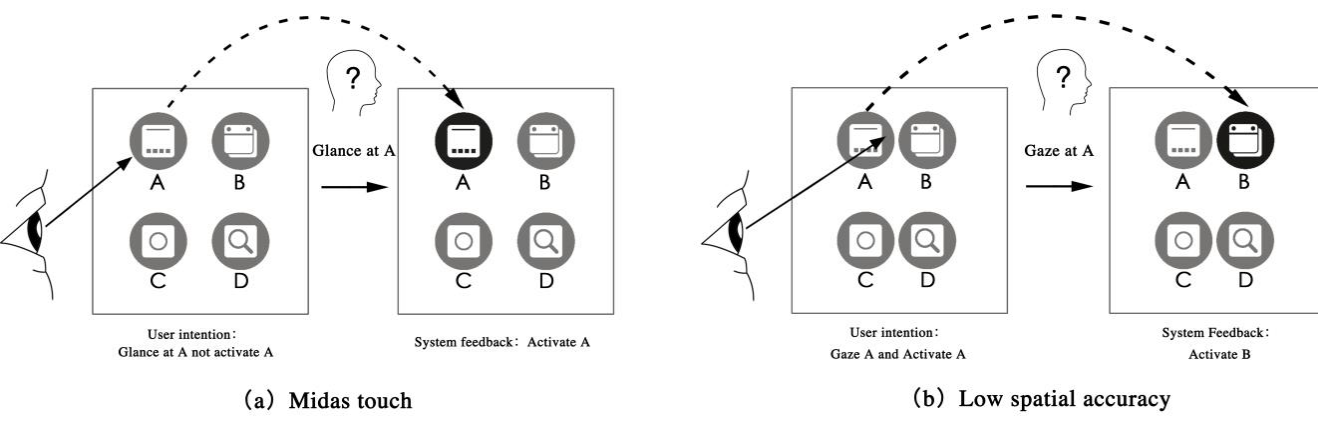
Midas touch (left) and low spatial accuracy (right).

The Eye–Computer Interaction (ECI) is an interactive method of controlling a computer or equipment by eye movements. An eye tracker is used to capture the user's line of sight data and identify the real-time position and trajectory of the gaze point. The ECI input commands include fixation, gaze gesture, blink, saccade, and smooth pursuit [1]. ECI has become the main control mode in the fields of head mounted displays (HMD) aiming system [2], and Artificial Intelligence [3], and it can also help patients with amyotrophic lateral sclerosis, hemiplegia, and pediatric cerebral palsy to communicate without obstacles. As the first interactive entrance, the user interface is one of the most important components in the ECI system, and all the ECI input commands are directly related to it. A good ECI interface design can improve user manipulation performance. Both, Windows and IOS operation systems have interface design specifications and standards [4, 5], but they are not entirely practical for the ECI system. There are two universal problems with the ECI system: “Midas touch” and “low spatial accuracy”. On the one hand, the ECI system cannot accurately distinguish whether the user is looking at a control for interaction or only getting information owing to the “Midas touch” [6, 7]. As shown in Fig. 1-a, the user’s original intention was to glance at A to get information but not trigger A; however, the system feedback result showed that A was triggered. On the other hand, “low spatial accuracy” resulted in a large deviation between the eye gaze position and the actual target control position, which led to a large probability of accidentally touching adjacent controls. As shown in Fig. 1-b, the user planned to gaze at A and trigger it, but adjacent control B was triggered instead.

In order to address these two questions, scholars proposed solutions in terms of the eye movement index [8, 9], the positioning calibration algorithm [10, 11, 12, 13, 14, 15], and multi-channels [16, 17]. However, these methods required a higher hardware configuration and algorithm accuracy, which brought new problems such as visual fatigue [18, 19] and a poor interaction experience [20, 21]. Therefore, in the interface design of the eye control system, it remains to be determined what kind of interactive feedback mode brings the highest interaction efficiency. In addition, we need to find out how large the interaction area is when the recognition rate is the highest? These are the two core issues of this research. The interaction efficiency can be evaluated comprehensively by the reaction time and user workload, and the recognition rate can be obtained by the accuracy rate or error rate.

Interactive feedback mainly involves eye-triggering movements and the dwell time. The main forms of eye-triggering movements are fixation, gaze gesture, blink, and closure, among which fixation is the most basic, widespread, and direct mode, so this research chose gaze as the triggering movement.

The interaction range mainly involves the size and location distribution of the functional control. The size refers to the spatial area of the control. The location distribution is mainly reflected in the saccade orientation or the speed of sight-lines' moving from the current triggering control to the next control. The faster the speed is, the greater the location advantage of the next control. The research on the best interactive feedback form and interactive range could reduce the Midas touch and improve the spatial accuracy to a certain extent.

**Figure 2:**
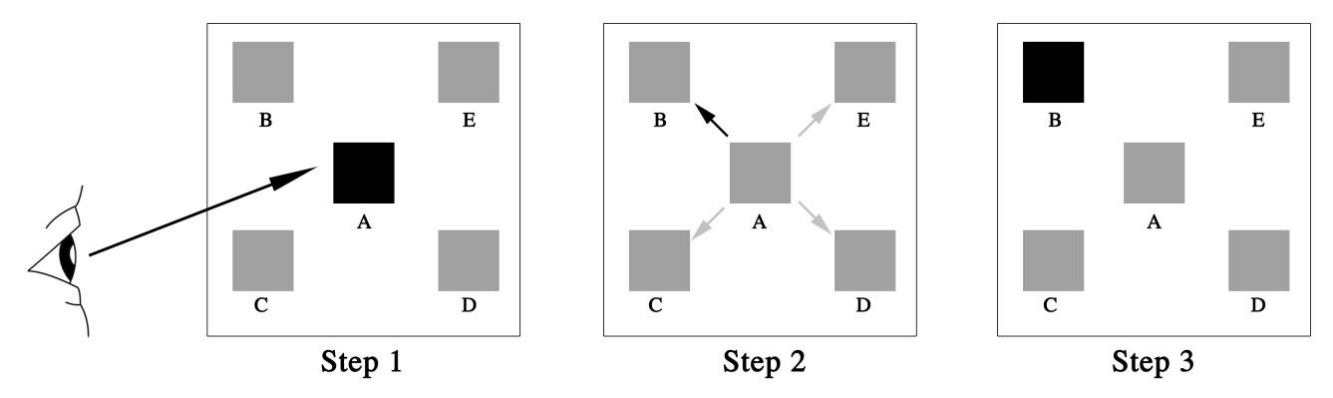
Basic gaze interaction model of the eye–computer interface (ECI) system.

This research mainly investigated the optimal gaze-triggering dwell time and size of functional controls. The gaze interaction basic model of the ECI system can be used to describe the process intuitively, as shown in Fig. 2. In Step 1, an individual gazes at module A and triggers it; in Step 2, a visual search is conducted to find target module B, and ignore other distractors; and in Step 3, an individual gazes at module B and triggers it. Steps 1 and 3 mainly involve the gaze-triggering dwell time, and Step 2 refers to the saccade orientation at the fastest saccade speed in different spatial positions. In Step 3, the black square is the functional control for triggering and gray is used in the non-triggering situation.

## Theoretic foundation of Experimental Series 1

### Theoretic foundation of shapes

Murata and Fukunaga's research on the size of ECI controls shows that square and circular controls are more efficient than 1:2 and 1:3 rectangles during interactions [22]. Thus, the controls were set to square in this study, and we also aimed to unify the spacing of controls.

### Theoretic foundation of size and position

According to a previous study, the size of controls is generally represented by the visual angle in the ECI system. The visual angle is the angle between the edge of a control and sight when the eye is looking at its center. It is also determined by the distance between the eyeball and the screen as well as the control’s size. Office man–machine instruction manual states that the distance between the eyes and the screen should be no less than 25 inches or 63.5 cm [23]. Feng and Shen suggested that the size of the trigger object should be no less than 1.5°, and the object spacing should be no less than 1.0° [24].

**Figure 3:**
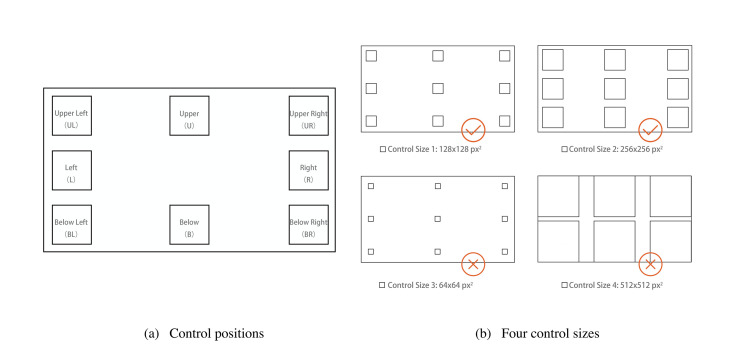
Schematic graph of the control position (left) and four control sizes.

Combined with the above research, the side length of an ECI control should be no less than 49 pixels, and the pitch should be no less than 33 pixels. According to The Windows Interface Guidelines for Software Design [4], this research selected four standard sizes as alternatives—64 × 64px^2^, 128 ×128 px^2^, 256 × 256 px^2^, and 512 × 512px^2^—which meet the requirements of the visual angle as well. Subsequently, the control size was further filtered according to other standards. The steps for screening selected control standards were as follows:

Nine square positions were set in experimental interfaces for placing controls, and these were evenly distributed to positions of 3 × 3. In Experimental Series 1, there was no control placed in the center position of the interface. Thus, control positions in the interfaces were named the upper left (UL), upper (U), upper right (UR), right (R), below right (BR), below (B), below left (BL), and left (L) giving a total of eight kinds, as shown in Fig. 3-a. Controls of different sizes appeared in the center of these eight areas in Experimental Series 1, and the gaps between the controls is not considered in this experiment.

For the control of size 512 × 512px^2^, the information capacity was only 6, and the spacing was relatively narrow. Users could obtain only 6 pieces of information too. This did not meet the requirements of the general interface information, so it was not selected.

In the process of sight recognition, the feedback point was changing in real-time. In the ECI system, the default range setting for sight was nearly 30 × 30 px^2^. If the control size was close to this value, the acquisition of key information in the target would be directly interfered with, resulting in a low interaction efficiency. Moreover, the phenomenon of sight drift is common in the ECI. When a control occupies a limited area, it is relatively difficult to trigger an action by dropping the viewpoint into the scope of the control for a certain period. The difficulty increases as the dwell time increases as well. Besides, the scanning speed of sight is extremely fast, which also leads to participants being unable to lock the target well if the control size is small, so the size 64 × 64px^2^ was not selected. Ultimately the sizes 128 × 128px^2^ and 256 × 256px^2^ were used (Fig. 3-b).

## Experimental Series 1

Experimental Series 1 was used as the pre-experiment. By recording the reaction time and accuracy rate under different control sizes, the optimal control size was screened, which paved the way for the formal experiment. The control size was set to two levels, and the position of control in the interface was set to eight levels. Experimental Series 1 adopted a single-factor, two-level experimental design.

There were 10 (repetitions) × 8 (positions) × 2 (128 × 128px^2^, 256 × 256px^2^) × 20 (participants), giving a total of 3200 trials in the whole of Experimental Series 1. For each single level (128 × 128px^2^, 256 × 256px^2^), 1600 sets of data were recorded. Each participant needed to complete 10 (repetitions) × 8 (positions) × 2 (levels) for a total of 160 trials, which took approximately 10 minutes.

### Participants

Twenty right-handed volunteers participated in Experimental Series 1 and 2. Their ages ranged from 21 to 25, the mean age was 23.1, and the standard deviation was 1.5. They were all undergraduate or postgraduate students from Southeast University. All participants were physically and mentally healthy, had no history of mental illness, and had normal or corrected vision without astigmatism. All of them had experience with using the Tobii eye tracker. The study protocol had been approved by the Southeast University Ethics Committee.

### Equipment

The computer system used was Windows 10, and the screen size was 1920 × 1080px^2^. The Tobii X2-30 Eye tracker is an eye movement tracker device with a 30 Hz sampling rate. It is small in size and was fixed at the bottom of the screen for the experiment. The device was used to get participants’ eye movement data during the process. The experimental platform was imported into the Tobii SDK installation package through Unity6.0 and compiled using C#.

### Experimental stimuli

The dependent variables were the reaction time and accuracy rate. The reaction time was directly output by the eye tracker and represented by t_a_, which referred to the length of time from the beginning of the trial to the trigger of control A. The accuracy rate was calculated as (number of tasks - number of failed tasks)/number of interfaces. Failed tasks included unintended activations and timeouts. When the residence time of a trial exceeded 10 seconds, the system counted it as a timeout automatically.

### Procedure of Experimental Series 1

**Figure 4:**
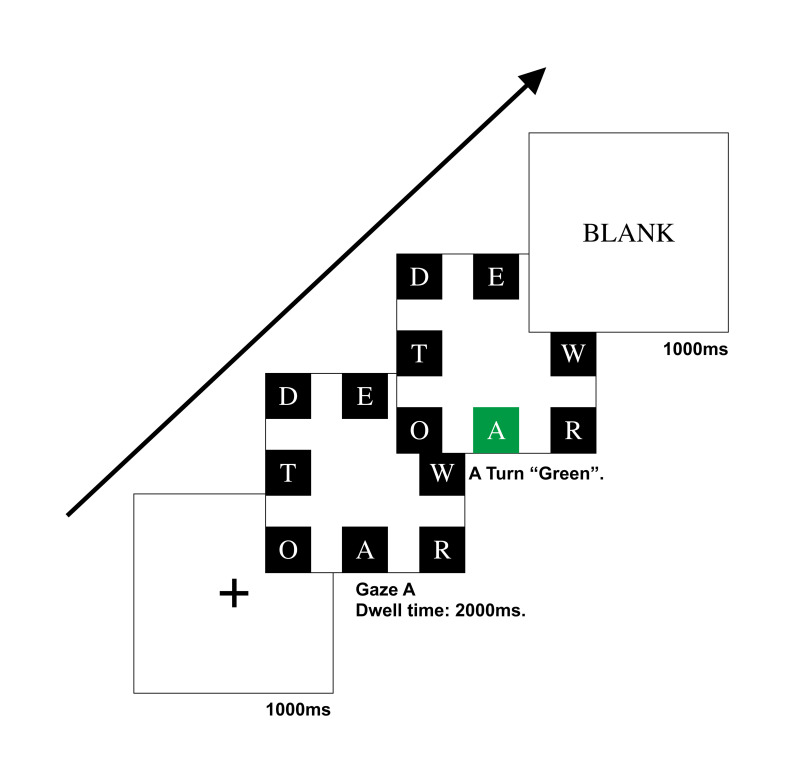
Flow chart of the Experimental Series 1 trial.

Participants were told to sit in front of the screen, with their eyes approximately 640 mm from the screen. The angle between the sight and the screen was 27° horizontally and 17° vertically. Experimental Series 1 trial began with a black cross focal point with a white background in the center of the screen for 1000 ms. Then, controls of different sizes with eight white letters on black backgrounds were displayed in the eight different directions of the white background screen. The eight different blocks used in each trial were random but contained control A each time. Participants were asked to find control A and gaze at it for 2000 ms until it turned green (#009944). When participants gazed at other controls but not control A, the related control turned red, which meant that participants were making wrong decisions. As participants finished the task with the right decision or could not finish in 15 seconds, a white blank display subsequently appeared for 1000 ms. Visual persistence was eliminated, and participants could take a rest as well. The procedure of Experimental Series 1 is depicted in Fig. 4.

All levels of independent variables were included in each round of experiments, and each level had one trial in a total of 40 rounds. Each participant needed to finish two rounds of experiments. After the first round of experiments was completed, there was a rest period. Trials appeared randomly.

### Data analysis of Experimental Series 1

#### 1 Reaction time

**Figure 5:**
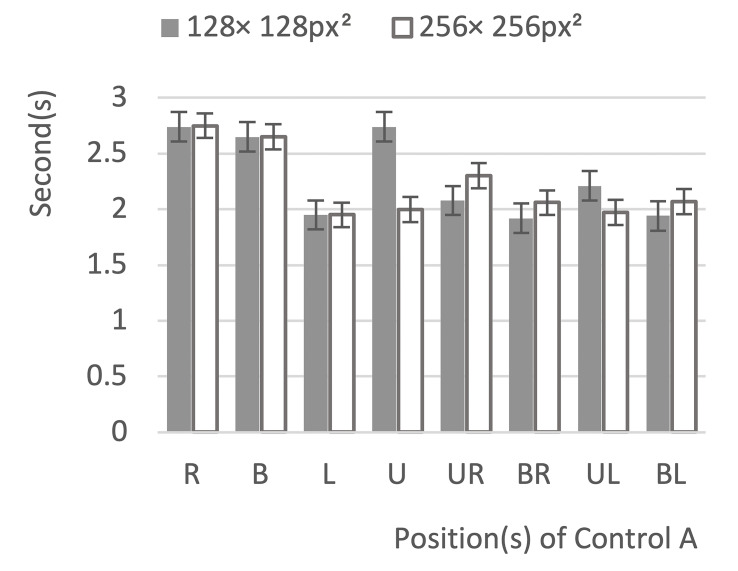
Box plot of reaction time.

In the data preprocessing, 20 data points had been removed corresponding to timeout failure and accidental fixation, the average time taken for task completion is shown in Fig. 5.

The graph shows that the reaction time of both 256 × 256px^2^ and 128 × 128 px^2^ was around 2 seconds. The unequal variance analysis of the independent samples T tests was used for data analysis. The results shows that there was no significant difference between the reaction time at the two size levels (p = 0.057, p > 0.05), that is, reaction time could not be used to select the optimal control size.

#### 2 Accuracy rate

**Figure 6:**
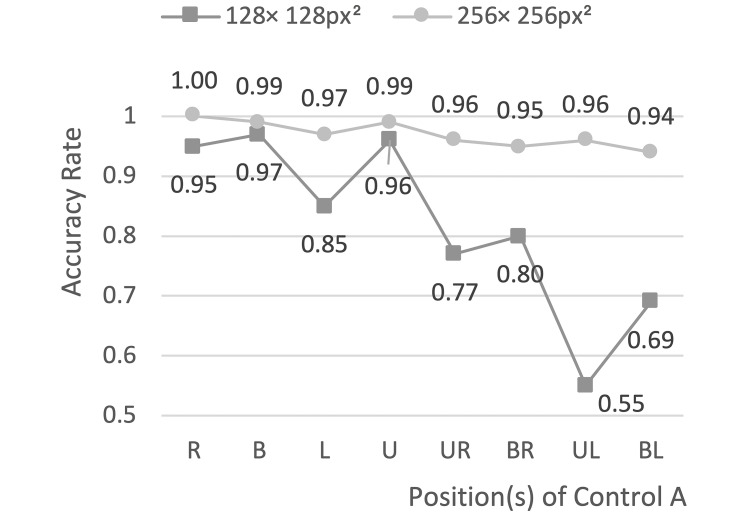
Line chart showing the results of the accuracy analysis.

An One Way ANOVA analysis was performed on the accuracy rate data (Fig. 6). The analysis suggested that there is a significant difference in the accuracy rate under different control sizes. The accuracy rate of the control with a size of 256 × 256px^2^ (0.97) was significantly higher than that of the control with a size of 128 × 128px^2^ (0.82) (F = 3.97, p<0.001). Therefore, 256 × 256px^2^ was chosen as the control size.

### Discussion of Experimental Series 1

In Experimental Series 1, the size levels were 128 × 128px^2^ (3.36° × 3.36°) and 256 × 256px^2^ (6.63° × 6.63°), respectively. Two dependent variables were adopted to select the optimal size: reaction time and accuracy rate. The average reaction time under levels of 128 × 128px^2^ and 256 × 256px^2^ were 2.05 and 2.38 s, respectively. According to the variance analysis, the size level had no significant influence on the reaction time. The short review above shows that the reaction time cannot be used as an effective indicator in this experiment. However, as can be seen from Fig. 5, the reaction time at level 128 × 128px^2^ was longer than that at level 256 × 256px^2^ in most cases. At the same time, there were certain differences in the response for different control positions. However, the impact of control positions on the efficiency is not discussed in this research.

Itakura and Sakamoto [25] built experimental interfaces with two different control sizes, with the width of the controls being 4° and 6°, respectively. In their study, the accuracy rate was calculated by deviation. If the deviation in one gaze was greater than 2°, it was considered a triggering failure. Finally, the accuracy of the interfaces was 96.7% and 88%, in which the control size made the difference. In Experimental Series 1, the accuracy rate of the 256 × 256px^2^ size was significantly higher than that of the 128 × 128px^2^ size (p < 0.05), which was consistent with the conclusion of Itakura et al. and was also consistent with the interaction suggestions proposed by Chitty [26]: in the ECI system, control sizes should be as large as possible, while the information capacity and fault tolerance should be also considered.

In addition, the less accurate areas in the interface were at the lower right corner, and the distribution area of the viewpoint before fitting was 225×183px^2^ [27]. In Experimental Series 1, all eight controls were located at the edge of the screen. Compared with the controls located in the center, the gaze accuracy of the controls at the lower edge and right edge was significantly reduced (p < 0.05), maybe this is related to the precision of the eye tracking device [28]. In order to ensure the accuracy and efficiency of the gaze input, the optimal size of the control located at the edge of the screen should be slightly larger. This conclusion supports the conclusion of experiment 1.

Fitz's law for ECI is as follows: when human eyes scan an object, the viewpoint will first move to the direction close to the target by a large distance and then be adjusted slowly and slightly through a small distance, before being positioned at the target [29]. The first stage of the saccade is quick. However, when the control is relatively small, the slow adjustment in second stage will last longer, which can affect the reaction time to some extent. Murata, Konishi, Moriwaka, and Fukunaga [30] verified the influences of the shape, area, and position of gaze-input controls on the reaction time (pointing time) in the ECI system and how it fits with the modified Fitz’s law model. Ware and Mikaelian [31] also fitted Fitz’s law to the reaction time model and obtained a relatively ideal result:

(1) $$\text{Pt}=a+b\times \log_{2}(d/s+1)$$

*Pt* refers to the reaction time, *d* is the distance between control B and the center of control A, *s* is the area of control B, *a* and *b* are constants, and log_2_ (*d* / *s* + 1) is defined as the difficulty. In the study of Ware and Mikaelian [31], the data of square controls fit the modified Fitz’s law best. This suggested that the reaction time of square controls would decrease significantly with the increase of control size. This is another main reason why we chose square as stimulus material (the first reason had been mentioned in the part of “Theoretic foundation of shapes”).

The purpose of Experimental Series 1 is to screen the optimal control size. In order to get the optimum gaze-triggering dwell time of optimal control size, Experimental Series 2 was conducted.

## Theoretic foundation of Experimental Series 2

Feng Chengzhi [24] suggested that the dwell time of the gaze trigger should be 500 ms. Helmert et al. [32] compared the performance of the virtual keyboard in gaze-input typing when the dwell time was 350, 500, and 700 ms after considering the KSPC (Key Strokes Per Character) and the character input speed. The results showed that 500 ms was the best solution. In Helmet research, there was a given task, while the present research is more or less free of any task context. The dwell time setting of gaze control is different according to task, such as gaze typing, control of an interface and other tasks.

The study of Graf and Krueger [33] showed that the length of the gaze was divided into two types: long fixations (>320ms), named the voluntary gaze, and short fixations (<240 ms), named the involuntary gaze, respectively. Therefore, the lower limit of the dwell time needs to be set at more than 200 ms so as to avoid the phenomenon of accidental fixation. According to Sibert, Linda, and Jacob [34], human eyes usually stabilize the viewpoint on the target object within 200–600 ms after a saccade. In their relevant studies, the dwell time was set to 200 ms. Therefore, based on previous research and human physiological characteristics, Experimental Series 1 locked the dwell time at 200–800 ms and set the step size at 200 ms.

## Experimental Series 2

Experimental Series 2 was used as the Main Experiment to investigate the optimum gaze-triggering dwell time by conducting a comprehensive evaluation of the accuracy rate, reaction time, and NASA-TLX (Task Load Index). According to the results of Experimental Series 1, control sizes A and B were set to 256 × 256px^2^. The purpose of setting control A was to make participants’ eyes start from the center of the screen uniformly to eliminate the original error. Control B was used as the interactive control. In this experiment, a single factor four-level design was adopted. Since the design of Experimental Series 2 was based on the conclusion of Experimental Series 1, same participants completed the two experiments at different time intervals of one week. The participants and equipment used in Experimental Series 2 were same as those used in Experimental Series 1.

There were 10 (repetitions) × 8 (eight positions) × 4 (200, 400, 600, and 800 ms) ×20 (participants), giving a total of 6400 trials in the whole of Experimental Series 2. For each single level (200, 400, 600, and 800 ms), 1600 sets of experimental data were recorded. Each subject needed to complete 10 (repetitions) ×8 (positions) ×4 (levels), giving a total of 320 trials, which took approximately 20 minutes.

### Experimental stimuli

**Figure 7:**
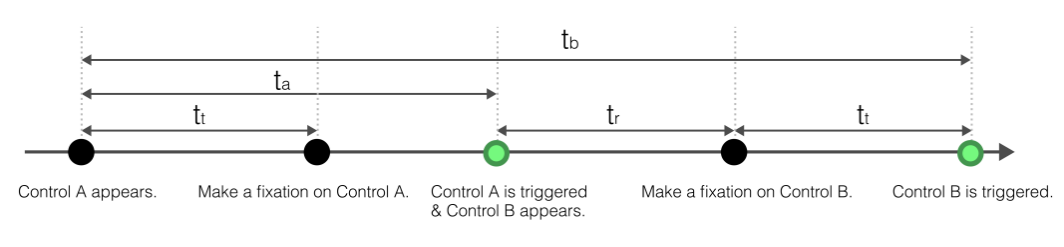
Timeline of a trial in Experimental Series 2.

Experimental Series 2 adopted a single factor four-level experimental design. The independent variable was set as the dwell time, which was the within-subject factor. The reaction time, accuracy rate, and subjective evaluation were selected as the criteria to select the dwell time. The reaction time refers to the time taken from seeing control B to pressing control B to react successfully. Each trial consisted of four periods:

t_a_: The time from the appearance of the interface to the trigger of control A;
t_*b*_: The time from the appearance of the interface to the trigger of control B;
t_*t*_: The time required for the control to be triggered, which was 200, 400, 600, or 800 ms respectively. It was equal to the level of triggering time foreach group.
t_*r*_: Reaction time.

t_a_ and t_*b*_ were the original data output by the eye tracker. A timeline of the duration of each section is shown in Fig. 7. The reaction time equals the total time minus the dwell time:

(2) $${\text{t}}_{r}=t_{b}-t_{a}-t_{t}$$

### Procedure of Experimental Series 2

The process was divided into four steps. The first and last steps were consistent with Experimental Series 1. In the second step, control A with a black background was displayed in the center of the white background screen. Participants needed to gaze at control A for a dwell time of 200, 400, 600, or 800 ms. When participants gazed at control A in the required time, control A would turn green （#009944）, which meant that control A was triggered. In the third step, control B with a black background was displayed in one of eight directions on the screen in a random order, and control A turned the original black color again. Meanwhile, participants needed to find control B and gaze at control B for a dwell time of 200, 400, 600, or 800 ms. In the fifth step, control B turned green.

**Figure 8:**
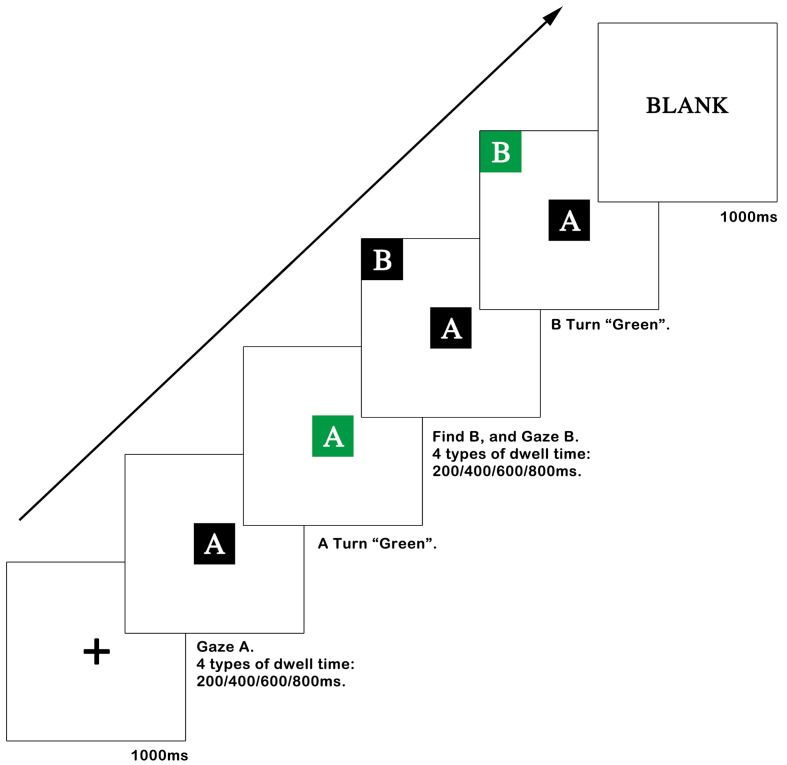
Flow chart of trials in Experimental Series 2.

All levels of independent variables were included in each round of experiments. Trials appeared randomly. Each participant needed to finish two rounds of experiments. After the first round of experiments was completed, there was a rest period. The procedure used for Experimental Series 2 is depicted in Fig. 8.

### Data analysis of Experimental Series 2

#### 1 Reaction time

In the data preprocessing, 24 data points had been removed corresponding to timeout failure and accidental fixation. In order to verify the specific impacts of different dwell times on the reaction time, SPSS was used for the following analysis.

**Figure 9:**
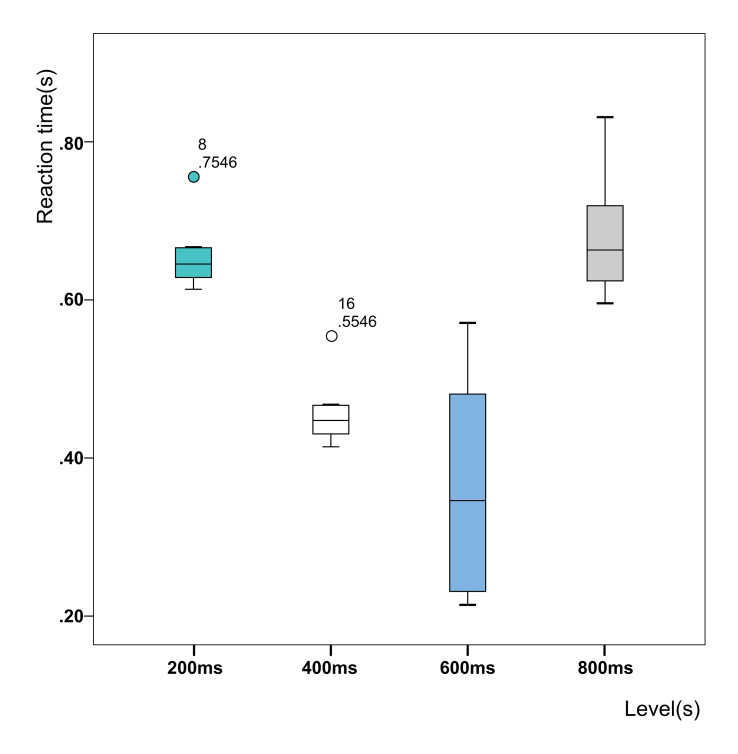
Boxplot of reaction times at different levels of dwell time.

A one-way analysis of variance was conducted on the dwell time, and it was found that the dwell time had a significant effect on the reaction time (p = 0, p < 0.05). The mean and standard deviation of the reaction time were then plotted as a boxplot (Fig. 9). The average reaction time at four trigger time levels was represented by $\bar{t}$ · ${\bar{t}}_{r200}$ = 0.656 s, ${\bar{t}}_{r400}$ = 0.456 s, ${\bar{t}}_{r600}$ = 0.362 s, and ${\bar{t}}_{r800}$ = 0.680 s , respectively. The comparison shows that ${\bar{t}}_{r600}$ was relatively small. However, the variance in the dwell time at 600 ms was 0.2, which was greater than the variance of the other three levels (0.002, 0.002, and 0.006). This indicates that at a level of 600 ms, the individual difference in dwell time between participants was large, which made the dwell time increase greatly between 0.55 and 1.1 s.

In order to further verify the inter-group differences in reaction time between the four levels, a post-hoc comparison was conducted. Since the variances of the four groups of data were different, Games–Howell analysis method was adopted without assuming homogeneity of variances. In this method, when p is less than 0.05, there is a significant difference between the levels. The results are shown in Tab. 1, where *p* values are given. As can be seen from the table, among the four dwell time levels:

**Table 1:** Post-hoc comparison of the reaction time between groups at four dwell time levels.

Reaction time			Sig.
Games-Howell	200 ms	400 ms	.000
		600 ms	.002
		800 ms	.000
	600 ms	800 ms	.001

There were significant differences between the 200 ms and 400 ms groups (p = 0, p < 0.05);

There were significant differences between the 200 ms and 600 ms groups (p = 0.002, p < 0.05);

There were significant differences between the 200 ms and 800 ms groups (p = 0, p < 0.05).

That is, in Experiment Series 2, levels with longer reaction times were the 200 ms and 800 ms conditions, and levels with shorter reaction times were the 400 ms and 600 ms groups. After comparing the average values, the optimal dwell time level of the control under the experimental conditions was selected to be 600 ms.

#### 2 Accuracy rate

The average accuracy rate was represented by $\bar{\text{g}}$. By calculation, the value of $\bar{\text{g}}$ at the four dwell time levels was ${\bar{\text{g}}}_{r200}$ = 100%, ${\bar{\text{g}}}_{r400}$ = 100%, ${\bar{\text{g}}}_{r600}$= 97.5%, and, ${\bar{\text{g}}}_{r800}$ = 98.1%, respectively—all basically above 95%. Based on the analysis of the variance of data, it was found that the dwell time had no significant influence on the accuracy rate of the task (p = 0.31, p > 0.05), that is, the accuracy rate had no obvious influence on the dwell time.

### NASA-TLX Evaluation

Subjective evaluation is an important criterion in interface design. In order to verify the results of data analysis and also evaluate the usability of the interface, for Experimental Series 2, the NASA-TLX scale [35] was used to carry out the subjective evaluation of the task load under different dwell time levels of the controls. Mental Demand (MD), Physical Demand (PD), Temporal Demand (TD), Operational Performance (OP), Effort (EF), and Frustration (FR) were used in the scale.

After confirming the questionnaire was matched with the experimental level by participant, each participant needed to fill out one questionnaire based on NASA-TLX scale for the related dwell time level in real time, four questionnaires in total. When each round experiment is over, Participants were asked to report their subjective feelings and feedbacks. After finishing the experiment, the six indicators were scored by 20 participants according to their experimental experience. In the score setting, full score is 100, a step length is 5, there are 20 scoring values in total. Participants needed to compare the above six indicators in pairs and selected the ones with a greater impact on the evaluation. The selected frequency of each indicator was counted to calculate the weight of it. The proportion of the corresponding frequency of each indicator in the total number of times was the weight of the indicator. Finally, the weighted average scores of six indicators were used to calculate the average load values, which were compared to select a level with a lower load value. Six indicators in the scale were divided in pairs, which were divided into 15 groups.

**Figure 10:**
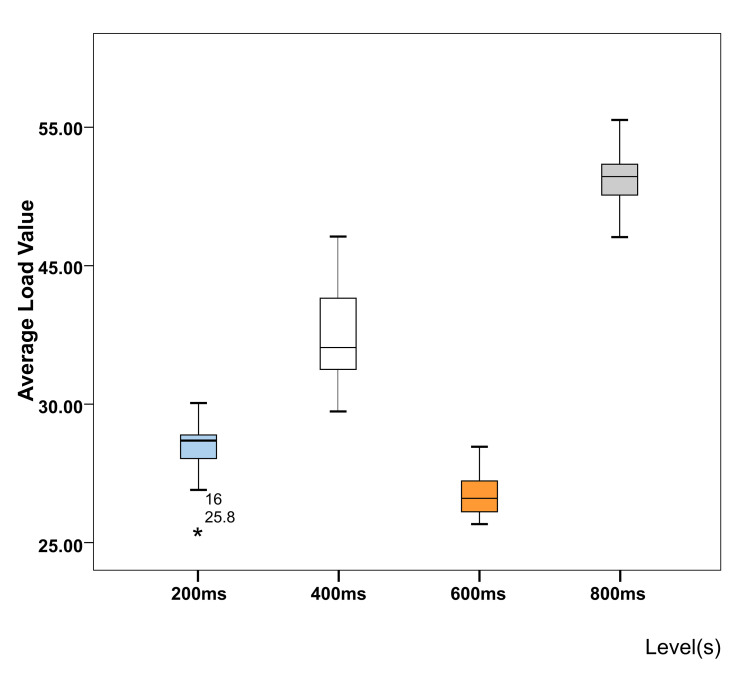
Average score of the task load at four levels.

The result shows that the weight of Mental Demand (MD) was 0.01, that of Physical Demand (PD) was 0.33, that of Temporal Demand (TD) was 0.13, that of Operational Performance (OP) was 0.20, that of Effort (EF) was 0.29, and that of Frustration (FR) was 0.04. After summarizing the scores, the average load value of the subject could be obtained by the weighted average scores of each indicator (Fig. 10).

Through calculation, it was determined that when the dwell time was 600 ms, the average load value of the participants was the lowest, 28.55, far lower than the average load value when the dwell time was 400 ms (40.05) or 800 ms (51.02), and slightly lower than the average load value under dwell time of 200 ms (31.87).

### Discussion of Experimental Series 2

The accuracy rate at the four dwell time levels was basically above 97%. The possible reason for this is that the tasks were not difficult, which caused a "ceiling effect". That could have meant that the accuracy rate had no distinguishing effect on the experiment. A similar pattern of results was obtained in the study by Itakura and Sakamoto [25]. They suggested that with a control width of 6°, the accuracy rate of the gaze trigger was basically higher than 95%, and there was no position discrimination. After analysis, the possible reasons were determined to be as follows:

In Experimental Series 2, the efficiency of the interface reached a high level by selecting factors such as size and shape. Therefore, most of the participants were able to complete the experimental task, so the accuracy rate had no differentiation effect. The same participants joined in two experiments, so experiments were designed as single post-test groups, that means participants were trained to operate skillfully after Experiment 1, which would have caused a certain amount of interference in the accuracy of the results. To some extent, the experimental results are influenced by the learning effect.

According to the traditional WIMP (Window/ Icon/ Menu/ Pointing device) interface specification while considering the instability of sight, the trigger was set to require one click instead of a double-click. Each trial included two one-click trigger tasks with a single type of trigger. The size, color, and shape of the controls in the interface was the same, without distinction. Therefore, the overall difficulty was relatively lower, which led to a high accuracy rate.

As for the dwell time, the data revealed a significant effect on the reaction time (p = 0, p < 0.05). Among them, the difference between groups at levels of 400 and 600 ms was relatively small and that between groups at levels of 200 and 800 ms was also relatively small. In addition, the average dwell time at the level of 600 ms was small (0.362 s). Finally, the optimal dwell time was selected to be 600ms. Zhang et al. [13] also found that during gaze-input interaction, the dwell time had a significant impact on the reaction time and task load. Finally, Zhang suggested that the dwell time under the level of 600 ms was the shortest with the least error.

When the dwell time is set to be relatively short, under a certain environment and task load, the duration of the subject’s gaze activity aiming to obtain information may reach or exceed the dwell time, resulting in accidental-touch, which has an impact on the reaction time. Meanwhile, the Midas touch is not well resolved.

Also, participants may reduce the scope of a single saccade and increase the number of saccades in order to avoid the occurrence of accidental fixation. This behavior may have an impact on the reaction time. Moreover, it may also increase the task load.

In a total of 320 task scenarios, each scenario contained two gaze triggers, one each for controls A and B. It was suggested that the dwell time affects the saccade efficiency after the eye gaze; hence, the dwell time of control A may have an impact on the saccade efficiency from controls A to B, which is manifested in an increased reaction time.

### Discussion of NASA-TLX Evaluation

In the evaluation part of Experimental Series 2, the NASA-TLX scale was used to collect the load information from participants. According to the statistical results of six indicators, the following analysis was made:

The weight of MD is small or even negligible. Based on this result, the following hypotheses were developed: the result may have been caused by the low difficulty of the task during the experiment. When carrying out activities with clear definition and single structure, participants may have thought that the mental load was small, meaning that the task was "very simple".

FR refers to a measure of frustration when a single task takes too long or the participant fails too many times, which was weighted only 4%. One of the possible reasons was that participants needed to finish more tasks in the NASA-TLX Evaluation Experiment, which was conducted 320 times. The number of tasks may dilute the frustration caused by a single task failure or a long reaction time. In addition, the accuracy rate was basically higher than 97%, and the number of failed tasks was small. Therefore, the proportion of frustration caused by the low accuracy of a single task was relatively small.

The proportions of PD and EF were 33% and 29%, respectively, which may be attributed to the fact that during the task, the participants' heads needed to be fixed for as long as possible to avoid errors during the eye tracking process. In addition, undergoing triggering and saccade movements while keeping the head fixed would have accelerated fatigue in participants.

After calculating the final score of the scale, we found that when the dwell time was 600 ms, the task load was the lowest (Fig. 10). According to the original data, at the level of 200 ms, scores of Physical Demand (PD) and Effort (EF) were higher than those at the level of 600 ms, while the other four indicators were lower than those at the level of 600 ms.

According to the feedback from participants, possible reasons for this result are as follows: when the dwell time was 200 ms, there was a frequent sight drift, and participants needed to spend time stabilizing their sight, so they tended to give higher Physical Demand (PD) and Effort (EF) scores. Considering the comprehensive efficiency and task load, 600 ms should be recommended as the optimal dwell time.

At the beginning of the NASA-TLX Evaluation Experiment, participants had to make a pairwise comparison. The benefit of this weighting is that it increases the sensitivity of the score to variables and reduces the differences among participants to some extent [37]. Three indicators in the scale were related to individuals: MD, PD, and TD. Three others were related to personal demands: OP, FR, and EF. Nikulin et al. [38] found that the requirement for MD was generally low during the execution of a specific task of a project. This conclusion is consistent with the conclusion of this research on MD.

In terms of total scores, Grier [39] collected a large number of NASA-TLX scales and concluded that, when the application fields of the scale were not differentiated, 80% of the total task load scores fell between 26.08 and 80.00. In the field of visual search, the median score was 57.89. The scores collected in the evaluation experiment ranged from 25.8 to 53.98, of which the minimum average score was 28.5, basically located near the minimum task load score in the same field. Therefore, the total score for this evaluation was relatively low. The possible reason for this is that in Experimental Series 1 and 2, the trigger type was only gaze-input and the controls had the same appearance, so participants needed less judgment. Therefore, the difficulty of the task was relatively small.

Bonnet et al. [40] studied the difference in task load in the states of free saccade and intentional search and found that the task load during intentional search was much larger. In addition, Recarte, Perez, Conchillo, and Nunes [41] found that the number of visual detections in the interface was negatively correlated with MD. These results are consistent with the conclusions of this research.

## Conclusion

This study explored the influences of the control size and dwell time of the gaze-triggered control on the inter-action efficiency of the ECI interface.

Through Experimental Series 1, the optimal control size was determined to be 256 × 256px2. The conclusion of Experimental Series 1 can be applied to the icon design of the Windows ECI operation system.

In Experimental Series 2, when the dwell time was 400 or 600ms, the interaction efficiency of participants was relatively high. Through the NASA-TLX scale, a dwell time of 600 ms has high efficiency and low task load. The result of Experimental Series 2 can help designers and engineers to optimize the interface design and develop systems with higher user experience and performance.

## Future work

There are still some deficiencies in this research:

The purpose of the Experimental Series 1 was to screen the optimal control size for further research, so the number of controls was limited to eight, and the controls had an equidistant distribution. Moreover, the appearance of controls was singular. Except for being used as a pre-experiment, the applicability of the results to other interface designs with different properties and structures has yet to be confirmed. In addition, in this interface, the evaluation of the size may have been affected by size and spacing, which should be further discussed in the future.

Many achievements have been made on the influence of the control position on the interaction efficiency, such as those shown in the study of Murata et al. [30]. However, in our study, the data analysis of Experimental Series 2 did not consider the influence of the control position and the difference between participants in terms of the reaction time. Further studies should be carried out in this area.

## Ethics and Conflict of Interest

The authors declare that the contents of the article are in agreement with the ethics described in http://biblio.unibe.ch/portale/elibrary/BOP/jemr/ethics.html and that there is no conflict of interest regarding the publication of this research.
